# Translation, Cross-Cultural Adaptation and Validation of Movement Behaviour Questionnaire into Malay Language (MBQ-M) for Measuring Movement Behaviors among Preschool Children in Kelantan, Malaysia

**DOI:** 10.3390/healthcare11091276

**Published:** 2023-04-29

**Authors:** Mohamad Hazni Abd Rahim, Mohd Ismail Ibrahim, Azriani Ab Rahman, Najib Majdi Yaacob

**Affiliations:** 1 Department of Community Medicine, School of Medical Sciences, Universiti Sains Malaysia, Kubang Kerian 16150, Malaysia; 2 Unit of Biostatistics and Research Methodology, School of Medical Sciences, Universiti Sains Malaysia, Kubang Kerian 16150, Malaysia

**Keywords:** translation, cross-cultural adaptation, movement behaviors, Malay translation, preschools

## Abstract

Measuring movement behaviours such as physical activity, sedentary behaviour, and sleep throughout 24 h is critical for assessing early childhood development. A valid tool based on cultural adaptation is required to achieve an appropriate result. Thus, this study aims to translate, culturally modify, and validate the movement behaviour questionnaire (MBQ) into Malay (MBQ-M) for preschool children in Kelantan, Malaysia. Permission to translate was obtained and the MBQ was translated using a ten-step process. Ten independent experts evaluated the content validity in terms of the content validity ratio (CVR), scale-level content validity index-average (SCVI-average), item-level content validity index (I-CVI), and the modified kappa statistic. The original questionnaire had modest adjustments: CVR 0.91, SCVI-average 0.93 for clarity, 0.95 for simplicity, and 0.93 for ambiguity. The SCVI-average relevance was 0.95, and the majority kappa value was excellent (>0.74). All the data imply that the overall content validity of the MBQ items is appropriate. Thirty parents assessed face validity, and the scale-level face validity index (S-FVI/Ave) for clarity (0.95) and comprehension (0.95) was satisfactory. In conclusion, the MBQ-M has satisfactory and acceptable content validity and face validity. Thus, it can be used as a valid tool to measure 24-h movement behaviours among preschool children in Malaysia.

## 1. Introduction

24-Hour movement behaviours, such as regular physical activity, adequate sleep, and minimizing time spent sedentary, are essential for the process development of early childhood [1]. The WHO defines early childhood as the time from birth to age eight [2], a period of rapid physical and cognitive development, and a time when a child’s habits are formed and lifestyle routines are open to changes and adaptations. The concept of 24-h movement behaviours is a new approach that explores how physical activity, sedentary behaviour, and sleep contribute to the healthy development of children and adolescents [3]. Traditionally, these behaviours have been studied in isolation, but recent research provides compelling evidence that the integrated relationship of movement behaviours should be considered [4]. Therefore, little is known about the interactive nature of movement behaviours and their importance to healthy development in preschool-aged children who have shown developmental advantages.

Movement is an important determinant of obesity and other health outcomes in children and adolescents. One of the world’s most challenging public health issues today is childhood obesity. It is estimated that about 39 million children under five years old are overweight or obese globally [5]. Countries with low to moderate income, such as Malaysia, are adversely affected by childhood obesity. In 2019, over half of the children under 5 who were overweight or obese lived in Asia [5]. The prevalence of overweight and obesity in Malaysia was 18.4%, which was much higher among preschoolers, or 1 in 5 children [6]. A recent study reveals that low physical exercise, excessive sedentary time, and insufficient sleep are the three most important risk factors for childhood obesity [7]. With increased physical activity, less sedentary behaviours (e.g., less screen time) and longer sleep, indices of physical health (e.g., lower BMI, obesity, cardiometabolic risk) and cognitive development (e.g., motor skills, academic) are positively related [8]. This is concerning because early childhood movement behaviours have consequences for health and well-being.

The World Health Organization recommends that children aged 3 to 5 spend at least 3 h of active play per day, in addition to 60 min of moderate to vigorous physical activity (MVPA), accumulate less than 60 min of sedentary recreational screen time, and sleep between 10 and 13 h per day [9]. A recent systematic review of the relationship between completing the 24-Hour Movement Guidelines and several health indicators revealed that only 5 to 24 percent of preschoolers fulfilled all three of the 24-h movement behaviour guidelines criteria [10]. Preschoolers’ age had significantly lower adherence compared with adolescents. The first study in Malaysia on movement behaviours has only 6.5 percent [11] adherence, and in other Asian countries, Hong Kong has 2.9 percent [12], both of which are among Asia’s lowest studies that meet all three movement guidelines criteria. However, there is little evidence about the proportion of Malaysian children meeting these recommendations. Currently, there is substantial variation in how movement behaviours are measured, and no validated assessment tools are culturally adapted to the Malay language.

Cross-cultural translation and validation need more than a literal translation to develop an identifying good questionnaire or instrument suitable to different target audiences of various cultures and languages [13]. Malaysia is a multi-ethnic nation with many languages and dialects spoken, but Malay (Bahasa Malaysia) is the most important language, serving as both the national and official language and as a lingua franca [14]. A thorough methodology for translating a measuring scale from one language to another is essential to ensure that the translated version is as similar to the original as feasible [15]. The MBQ is a validated rapid assessment tool developed by Trost [16] for measuring early childhood physical activity, screen time, and sleep. Parents or guardians will respond to the proxy self-reported questionnaires used to quantify 24-h movement behaviours. Identifying and fully understanding young children’s movement behaviours is crucial for early assessment in healthcare and the development of effective interventions to prevent childhood obesity and other health effects. To address these knowledge gaps, the current study aims to translate, culturally adapt, and validate the Movement Behaviors questionnaire (MBQ-M) for Malaysian families.

## 2. Materials and Methods

### 2.1. Translation Movement Behaviors Questionnaire (MBQ)

The MBQ is a validated tool for assessing movement behaviors in children less than six years old [16]. It contains a 15-item short-form instrument for measuring physical activity, screen time, and sleep in preschool-aged children. The MBQ assesses physical activity and screen time separately for weekdays and weekends, then calculates a weighted daily average for daily total active play, energetic play, passive screen time, and interactive screen time. The sleep was assessed in terms of sleep duration and sleep routine. There is an open-ended version (responses are provided in hours and minutes per day) and a closed-answer version (response options are selected within a range of time). The MBQ has moderate to excellent test–retest reliability, with intraclass correlation coefficients (ICC) ranging from 0.68 to 0.98 and 0.44 to 0.97 for the open- and closed-ended versions, respectively. Demonstrating concurrent validity between the 24-h diary (documented 24-h use of time for each child for screen time and sleep time) and MBQ screen time and sleep (r = 0.44–0.86), significant positive correlations were observed for both the open- and closed-ended versions; moreover, device-measured time in total active play and energetic play with MBQ physical activities have also been found to have positive correlations (r = 0.27–0.42) [16]. We used a closed-ended MBQ questionnaire, which simplified the cognitive task of summing estimates of screen time and physical activity from various modalities.

The translation process followed the established guidelines [17]. This guideline comprised ten stages, including:

(1) Preparation

The original form of the MBQ questionnaire was used with permission. The MBQ translators were identified, contacted, and informed of the study’s objectives at this point. During this phase, researchers, panel committees, and translators were established in line with the time frame of each stage.

(2) Forward Translation

The first translator was a Ministry of Health (MOH) psychologist and the second one was a medical doctor from a teaching university. Both translators were fluent in English and Malay. They noted any questions, phrases, or statements in the questionnaire that were difficult or impossible to translate. Each translator worked independently on their translations. As a translation aid, a digital copy of the MBQ was provided.

(3) Reconciliation

The panel committee evaluated both forward translations. A public health lecturer, a family health lecturer, a professional translator, a linguistics lecturer, a psychologist, and a statistician constituted the panel committee. Each phrase in each translation was compared during the reconciliation phase. Alternative terms or phrases were used in place of those that did not fit the cultural context of Malaysia. After the meeting, the review committee compiled and approved a common forward translation of the MBQ-M that will be used in the following phase. At this stage, ten independent experts also evaluated the questionnaire content validity.

(4) Backward Translation

The common forward translation of the MBQ-M was sent to two additional independent translators to translate back into English. Translator one was a university English teacher and translator two was a physician with public health qualifications. Both were different groups of translators from the forward translation process. The original questionnaire and the study’s objectives were concealed from the translators. Both translators were fluent in both languages.

(5) Backward Translation Review

The same review committee that synthesised the forward translation examined the backward translation review. They examined the equivalence in terms of both versions’ concepts, items, and semantics [18]. The conceptual equivalence assessment aimed to ascertain whether the understanding concept of physical activity, screen time, and sleep were present in both settings and acceptable to both populations. Item equivalence concerns whether the specific items are relevant and suitable in target populations [19]. The semantic equivalence of the MBQ between the original English version and the translated version in Malay has assured similarity of meaning through the process of forward and backward translation.

(6) Harmonisation

Harmonisation was done to ensure conceptual equivalence between the source and target language versions and between all translations by identifying and resolving potential translation conflicts that may arise between different language versions. This added a step for quality control and enabled the secure combination of trial data from around the world. The translation evaluation following the harmonisation took place at the same meeting. The review committee was generally pleased with the similarity between the English version of the MBQ and its common forward translation. Considering this, it was determined that the common forward translation would serve as the pre-final MBQ-M to be tested in the subsequent phase.

(7) Cognitive Debriefing

This phase identifies any conceptual errors and confusion among a relevant sample of respondents or laypeople. A pre-final MBQ-M cognitive debriefing was conducted on a sample of five parents with varying levels of education, ranging from primary school to postgraduate level. Each respondent who volunteered to participate was informed of the purpose and procedures of cognitive debriefing. Two men and three women between 34 and 45 years old responded.

Respondents were given 5 min to complete a pre-final MBQ-M. We inquired as to what respondents imagined when they first read or heard a word or phrase, and how they selected their responses. In addition, respondents were asked if there was a term or sentence they did not understand or found challenging. When there were multiple options for an item or statement, respondents were asked to select the option that corresponded most closely to their typical usage. On the back of their questionnaires, red-pen comments and suggestions were written by respondents. For each completed questionnaire, a test time was recorded.

(8) Review of Cognitive Debriefing Results and Finalisation

A comparison was made between the respondent’s interpretation of the translation and the original text to identify and correct any issues. The review committee members were briefed on the cognitive debriefing’s outcomes. The committee considered all comments and suggestions from respondents and adjusted as necessary.

(9) Proofreading

Proofreading was conducted to consolidate the MBQ-M and eliminate any typographical or grammatical errors. The committee felt that its presence was essential since it provided a critical chance to verify any small flaws were fixed prior to the instrument’s approval for usage among the intended respondent.

(10) Final Report

The entire translation process was detailed in a concluding report. This was done to comprehend the terminology decisions underlying the translation. This is crucial for ensuring consistency in future translations of the same measure in multiple languages.

### 2.2. Validation Movement Behavior Questionnaire-Malay (MBQ-M)

#### Content Validity

The process of content validation started in August 2022 with the appointment of a panel of ten independent experts from Malaysia’s east coast who were fluent in both languages. As suggested by the previous study, at least six experts should be involved in content validation, but no more than ten is considered necessary [20]. They had different professional backgrounds: two paediatricians, two preschool teachers, two medical doctors, one sports science Ph.D. candidate, one lecturer family physician, one public health physician, and one psychiatrist with a minimum of five years of field experience. The content was validated both in person and via email. The experts were asked to review the MBQ-M validity method, appropriate words, and content to ensure that the items were culturally appropriate for the Malaysian population.

Through the quantitative content validity method, the experts assessed the content validity ratio (CVR), item-level content validity index (I-CVI), scale-level content validity index-average (SCVI-average), and modified kappa agreement. The experts were asked to evaluate the items of the MBQ in terms of essentiality, relevancy, clarity (understandability), simplicity, and ambiguity by rating each item on a Likert scale. The essentiality of the item was assessed on a 3-point Likert rating scale (1 = not necessary, 2 = useful but not necessary, 3 = essential) to measure the CVR [21]. The content validity ratio varies between 1 and −1 and an essential rating of 3 was considered valid. The following formula determined the method of calculation for CVR:

CVR = (N_e_ − N/2)/(N/2)

where CVR is the Content Validity Ratio, Ne is the number of panellists indicating essentials about a specific item, and N is the total number of panellists. According to the Lawshe [21] table, for ten panellist members, a CVR greater than 0.62 was an acceptable CVR.

The CVI is an index of inter-rater agreement among experts. Relevancy, clarity, simplicity, and ambiguity assessments were assessed on a 4-point Likert rating scale as recommended by Yaghmaie [22] for measuring CVI as follows: scale relevancy (1 = irrelevant item, 2 = somewhat relevant item, 3 = a quite relevant item, 4 = highly relevant item), clarity scale (1 = not clear, 2 = item needs some revision, 3 = clear but needs minor revision, 4 = very clear), simplicity scale (1 = not simple, 2 = item needs some revision, 3 = simple but needs minor revision, 4 = very simple), and ambiguity scale (1 = doubtful, 2 = item needs some revision, 3 = no doubt but needs minor revision, 4 = meaning is clear). Items with a rating of 1 and 2 were considered invalid and items with a rating of 3 and 4 were considered valid.

CVI can be calculated both for item level (I-CVI) and scale-level (S-CVI). I-CVI represents the proportion of agreement on each item, which ranges from 0 to 1, whereas S-CVI is well-defined as the proportion of items rated content valid [20]. Item level, I-CVI, was calculated as the number of experts giving a rating of three or four of each item divided by the total number of experts, as shown in Table 1. The item will be approved if I-CVI ≥ 0.78 [22] and an SCVI-average of ≥0.90 indicates the item has acceptable content validity [23]. S-CVI can be calculated by S-CVI/average number of experts (Ave).

After calculating I-CVI for all instrument items and probability of chance agreement (PC), the modified kappa was calculated because it provides essential additional information about the degree of agreement beyond chance. The probability of chance agreement was first calculated for each item by the following formula:

Pc = [N!/A! (N − A)!] × 5^N^ (1)

where N = number of experts panel and A = number of panelists who agree on the item (rating of 3 and 4). After calculating I-CVI for all instrument items, kappa is computed by entering the numerical values of probability of chance agreement (Pc) and CVI of each item (I-CVI) in the formula as shown in Table 2:

K= (I-CVI − Pc)/(1 − Pc)(2)

Evaluation criteria for modified kappa are as follows: values above 0.74, between 0.60 and 0.74, and those between 0.40 and 0.59 are considered excellent, good, and fair, respectively [24].

### 2.3. Face Validity

The face validity index (FVI) is the degree to which a measure captures the intended construct in the view of intended participants. According to Lau, Yusoff [25], the acceptable sample size required for parents of preschool children for the face validation index was 30 samples. A convenience sample of 30 parents/guardian children aged four to six from a selected private preschool was recruited in September 2022 at Kota Bharu district, Kelantan. Parents or guardians aged 25–46 years from different education levels and occupational statuses were selected for pre-testing on face validation. Envelopes containing a copy of the informed consent, study information, and a self-administered questionnaire were given to selected parents. Parents or guardians agreed to participate, and the completed questionnaires were returned to the teachers by envelope via their children.

The face validation aimed to assess the translated items’ clarity and comprehensibility of respondents. Parents/guardians are required to rate clarity on a scale from 1 (not at all clear) to 4 (very clear) and comprehension on a scale from 1 (unable to understand at all) to 4 (very easy to understand). Item-level face validity index (I-FVI) is the proportion of raters giving an item a clarity or comprehension rating of 3 or 4. The I-FVI was calculated by the formula I-FVI = the raters in agreement on item divided by the number of raters. S-FVI/Ave is a scale-level face validity index based on the average method, calculated by the formula S-FVI/Ave = sum of I-FVI scores divided by a total number of items. In this study, the value of the face validity index (I-FVI) ≥ 0.8 and S-CVI/Ave ≥ 0.9 constituted a satisfactory level of face validity [25].

### 2.4. Ethical Consideration

The approval for this research was obtained from the Human Research Ethics Committee of Universiti Sains Malaysia (USM/JEPeM/22070491). The confidentiality of the data was strictly maintained. Only the author and supervisors had access to the available data. Later, the reporting and publications were carried out with no respondents’ names mentioned.

## 3. Results

### 3.1. Cross-Cultural Adaptation

Several modifications were made to the MBQ before forward and back translation to adapt it to Malaysian culture. In items Q1a and Q1b, the term “typical weekday” has been changed to “working day”, a commonly used word in Malaysia. The term “typically weekend” was added to items Q2a and Q2b because the definition of a weekend in Malaysia varies by state. For example, in northeast Malaysia, the weekend is Friday and Saturday.

The English term “screen time” was maintained in questions Q3 and Q4; there is no accurate Malay translation. FaceTime and Skype have been replaced with Google Meet and Microsoft Teams, popular among children, for questions Q5a through Q6b. The final two questions, Q5 and Q6, determined that the term “video console games” is unfamiliar to our population, and examples such as PlayStation, Nintendo, and Xbox were provided.

### 3.2. Content Validation

The content validation of the experts’ ratings and responses revealed that the MBQ-M contained significant and vital questions. All remaining MBQ-M items had a content validity ratio (CVR) greater than 0.62. I-CVI scores of 0.95 for relevance, 0.93 for clarity, 0.95 for simplicity, and 0.93 for ambiguity indicated adequate and acceptable content validity. Two items in Q2a and Q2b must be revised because their I-CVI clarity and ambiguity scores were less than 0.75. The items were modified in response to the suggestions of the panel experts and panel committee. The I-CVI determined that the majority of the items were appropriate and approved in terms of relevancy, clarity, simplicity, and ambiguity (Table 1).

As shown in Table 2, the modified kappa agreement demonstrated excellent inter-rater agreement on item ratings for most MBQ-M items (K range of 0.8 to 1.0 for excellent). For questions Q2a and Q2b, the evaluation showed good modified kappa values on clarity and ambiguity items (K 0.66).

### 3.3. Face Validation

The face validity index of clarity and comprehension were 0.95 and 0.93, respectively. The face validity index indicates a satisfactory and adequate level of face validity. The details of item-level indices are summarised in Table 3.

## 4. Discussion

In this study, the translation process follows the guidelines outlined by Wild, Grove [17] with the assistance of a panel of experts. This procedure ensures that the translated items retain their original meaning and that the translated questionnaire is free of confusion. According to our knowledge, this is the first study to translate and culturally adapt a measure of preschool children’s movement behaviors in Malaysia. The MBQ-M content and face validity in Malay were evaluated. Minor translation discrepancies were identified, addressed, and resolved, resulting in a final Malay version of the questionnaire.

The cross-cultural adaptation process focuses not only on translation but also on cultural adaptation that is appropriate for a new setting. Even within Malaysia’s vast geographical boundaries, the Malay language has a huge variety of accents, slang, and nuances. We had difficulty finding accurate Malay translations of words such as “screen time” during the reconciliation process. The panel committee and researcher agreed to include both the Malay term “masa skrin” and the English term “screen time” in items Q3 and Q4. The translation process may encounter difficulty when two different languages have nonequivalent words and may lead to different meanings [26]. It demonstrated that cross-cultural research translation was becoming essential in health research. Additionally, problematic items, such as those requiring responses related to frequency/likelihood, like “Typical” and “Of this time”, were modified based on suggestions for alternatives by the panel committee and reorganized Malay words for improved clarity. To prevent confusion, we separated questions about frequent behavior in the past week; working days or weekends were used as heading sentences.

In the cognitive interviews, parents understood most items but requested adjustments to organizing the questionnaire, wordiness, and recall time. Parents also emphasised the need to standardise the recalling period for all scales to “a regular day, considering the past week” and “defining working day and weekend”. In addition, a definition of “vigorous activity” was added to items Q1b and Q2b, which refers to expending a lot of energy. These adaptations highlight the need to conduct cognitive interview studies before new or adapted measurement scales are deployed in other cultural settings.

An important finding of the current study was the difficulty in estimating and accumulating screen time and physical activity over all five weekdays and two weekends. Previous research discovered the same issue when measuring estimated screen time using the same method [27]. In Q1–Q6, the sentence structure was improved, and repetitive questions were simplified. Parents also reported that the term “video console games” was confusing. Examples such as PlayStation, Nintendo, and Xbox were provided to minimise the inconvenience. This finding highlights the importance of rigorously testing and adapting measurement scales before using them in research populations with low numeracy and literacy levels.

Based on clarity, simplicity, ambiguity, and relevance items, this study indicates that the MBQ-M has good, clear, simple, and essential content validity. All measurement items provide a comprehensive view of content validity [22]. This demonstrated that the translation process was culturally appropriate and reliable for use with the intended population. The translated version must be stylistically appropriate and culturally acceptable to the intended population and have the same communicative effect as the original. It must show an acceptable and approved content validity index I-CVI/Ave of at least 0.90 [23] and CVR of 0.62 [21]. In this study, the MBQ-M demonstrated a CVI of 0.93 to 0.95 and a CVR of 0.91, indicating the content was highly significant to represent the measured outcome for the Malaysian population. In addition, the kappa statistic revealed that most items with a value greater than 0.74 provided crucial support for the CVI result, as it provides information about the degree of agreement that is beyond chance. Modifications were made to Q2a and Q2b, which were determined to require revision, to increase clarity and reduce ambiguity. Both items are related, and an example of light physical activity was added to item Q2a to differentiate between mild and moderately vigorous activity in item Q2b.

The back-translated version (from Malay to English) needed only minimal modifications, demonstrating clarity and the lack of inconsistencies and/or ambiguities. All 15 MBQ-M questions have excellent clarity and understanding of items, demonstrating that the MBQ has a high index of face validity. By assessing the clarity and understanding of the inventory’s language, any different researcher and participant views of the items’ structure were removed [28].

This study had a few limitations. The common issue of the translation process in the translation of English into the Malay version was the linguistic problem [29]. For example, the discrepancies in the translation of certain English axes into Malay resulted in the use of various words to express the same meaning. Even if the terms exist in Malay, they may not be appropriate or suitable for translation. In addition, several participants suggested modifications that could not be incorporated because the expert committee determined that they would fundamentally affect the objective of the original questions. However, such instances were few, and most of the parental feedback was implemented.

Finally, despite the above-mentioned limitations, our results support the use of the MBQ-M to evaluate 24-h movement behaviours in Malaysian preschools. Additional benefits of the MBQ-M include lower burden costs, applicability in a large sample population, and quick evaluation in close-by facilities for clinical assessment of childhood obesity, as examples. We advise additional research into the development, association, and associated health outcomes in young children. Childhood obesity and physical inactivity are well-known public health issues that are linked to several unfavourable health outcomes.

The results of these studies will benefit public health, particularly in Malaysia, in battling against the growing problems of childhood mental illness and obesity. Better child health outcomes, including improved physical and cognitive development, have been associated with longer sleep duration, increased physical activity, and limited screen time [30,31]. Additionally, because this study is primarily concerned with young children, it may be a reliable indicator of movement behaviours, contribute to the documentation of risks, and allow for earlier population-level intervention through more applicable and targeted programmes. The findings in this paper are part of a phase one research study at USM. The findings of the study will be helpful in the next phase, which will investigate the associations between movement behaviours and socioemotional difficulties in preschool children.

## 5. Conclusions

In conclusion, the MBQ-M content and face validity were both satisfactory. As a result, it is a reliable instrument for assessing 24-h movement behaviors in preschoolers in Kelantan, Malaysia. The validated MBQ-M can assist researchers of interest in evaluating children’s movement behaviors and ultimately coming up with better ideas for early intervention programs to improve the situation.

## Figures and Tables

**Table 1:** Content validity index of items relevancy, clarity, simplicity, and ambiguity, and content validity ratio of essentiality MBQ-M items.

Items MBQ-M	Relevancy			Clarity			Simplicity			Ambiguity			Essentiality		
	Ne ¹	I-CVI	Interpretation	Ne ¹	I-CVI	Interpretation	Ne ¹	I-CVI	Interpretation	Ne ¹	I-CVI	Interpretation	Ne ¹	CVR	Interpretation
**Q1a**	9	0.9	Approved	10	1	Approved	10	1	Approved	10	1	Approved	9	0.8	Remained
**Q1b**	9	0.9	Approved	10	1	Approved	10	1	Approved	10	1	Approved	9	0.8	Remained
**Q2a**	10	1	Approved	7	0.7	Need revision	9	0.9	Approved	7	0.7	Need revision	10	1	Remained
**Q2b**	10	1	Approved	7	0.7	Need revision	9	0.9	Approved	7	0.7	Need revision	10	1	Remained
**Q3a**	10	1	Approved	10	1	Approved	10	1	Approved	10	1	Approved	10	1	Remained
**Q3b**	10	1	Approved	10	1	Approved	10	1	Approved	10	1	Approved	10	1	Remained
**Q4a**	9	0.9	Approved	9	0.9	Approved	9	0.9	Approved	9	0.9	Approved	9	0.8	Remained
**Q4b**	9	0.9	Approved	9	0.9	Approved	9	0.9	Approved	9	0.9	Approved	9	0.8	Remained
**Q5a**	10	1	Approved	10	1	Approved	10	1	Approved	10	1	Approved	10	1	Remained
**Q5b**	10	1	Approved	10	1	Approved	10	1	Approved	10	1	Approved	10	1	Remained
**Q6a**	9	0.9	Approved	9	0.9	Approved	9	0.9	Approved	9	0.9	Approved	9	0.8	Remained
**Q6b**	9	0.9	Approved	9	0.9	Approved	9	0.9	Approved	9	0.9	Approved	9	0.8	Remained
**Q7**	10	1	Approved	10	1	Approved	10	1	Approved	10	1	Approved	10	1	Remained
**Q8**	10	1	Approved	10	1	Approved	10	1	Approved	10	1	Approved	10	1	Remained
**Q9**	9	0.9	Approved	9	0.9	Approved	9	0.9	Approved	9	0.9	Approved	9	0.8	Remained
**SCVI-average**		0.95		0.93			0.95			0.93			**CVR/** **Average**	0.91	

Item-level content validity index (I-CVI), ≥0.78 approved; Average item-level content validity index = average I-CVI, ≥0.90 approved. Ne ¹: Number of experts evaluated the item, CVR or Content Validity Ratio = (Ne − N/2)/(N/2) with ten persons at the expert panel (N = 10), the items with the CVR bigger than 0.62 remained; scale-level content validity index, average (S-CVI/Ave); content validity index average (CVR/Ave).

**Table 2:** Modified kappa agreement of the MBQ-M items.

Items MBQ-M	Relevancy		Clarity		Simplicity		Ambiguity		Essentiality		Interpretation
	Pc ¹	K ²	Pc ¹	K ²	Pc ¹	K ²	Pc ¹	K ²	Pc ¹	K ²	
**Q1a**	0.010	0.90	0.001	1.00	0.001	1.00	0.001	1.00	0.010	0.80	Excellent
**Q1b**	0.010	0.90	0.001	1.00	0.010	0.90	0.001	1.00	0.010	0.80	Excellent
**Q2a**	0.001	1.00	0.117	0.66	0.010	0.90	0.117	0.66	0.001	1.00	Good
**Q2b**	0.001	1.00	0.117	0.66	0.001	1.00	0.117	0.66	0.001	1.00	Good
**Q3a**	0.001	1.00	0.001	1.00	0.001	1.00	0.001	1.00	0.001	1.00	Excellent
**Q3b**	0.001	1.00	0.001	1.00	0.010	0.90	0.001	1.00	0.001	1.00	Excellent
**Q4a**	0.010	0.90	0.010	0.90	0.010	0.90	0.010	0.90	0.010	0.80	Excellent
**Q4b**	0.010	0.90	0.010	0.90	0.001	1.00	0.010	0.90	0.010	0.80	Excellent
**Q5a**	0.001	1.00	0.001	1.00	0.001	1.00	0.001	1.00	0.001	1.00	Excellent
**Q5b**	0.001	1.00	0.001	1.00	0.010	0.90	0.001	1.00	0.001	1.00	Excellent
**Q6a**	0.010	0.90	0.010	0.90	0.010	0.90	0.010	0.90	0.010	0.80	Excellent
**Q6b**	0.010	0.90	0.010	0.90	0.001	1.00	0.010	0.90	0.010	0.80	Excellent
**Q7**	0.001	1.00	0.001	1.00	0.001	1.00	0.001	1.00	0.001	1.00	Excellent
**Q8**	0.001	1.00	0.001	1.00	0.010	0.90	0.001	1.00	0.001	1.00	Excellent
**Q9**	0.010	0.90	0.010	0.90	0.001	1.00	0.010	0.90	0.010	0.80	Excellent

Pc ¹ = probability of chance occurrence [N!/A!(N − A)!] × 5^N^, K ² = Modified Kappa agreement. (I-CVI − pc)/(1 − pc). c Evaluation, kappa values: fair = 0.40–0.59 good = 0.60–0.74, excellent > 0.74.

**Table 3:** The face validity index of clarity and comprehension MBQ-M items.

Items MBQ-M	Clarity			Comprehension		
	Ne ¹	I-FVI	Interpretation	Ne ¹	I-FVI	Interpretation
**Q1a**	27	0.90	Approved	26	0.87	Approved
**Q1b**	28	0.93	Approved	28	0.93	Approved
**Q2a**	28	0.93	Approved	26	0.87	Approved
**Q2b**	30	1.00	Approved	26	0.87	Approved
**Q3a**	28	0.93	Approved	30	1.00	Approved
**Q3b**	30	1.00	Approved	30	1.00	Approved
**Q4a**	27	0.90	Approved	26	0.87	Approved
**Q4b**	27	0.90	Approved	26	0.87	Approved
**Q5a**	28	0.93	Approved	28	0.93	Approved
**Q5b**	28	0.93	Approved	27	0.90	Approved
**Q6a**	27	0.90	Approved	28	0.93	Approved
**Q6b**	28	0.93	Approved	28	0.93	Approved
**Q7**	30	1.00	Approved	30	1.00	Approved
**Q8**	30	1.00	Approved	30	1.00	Approved
**Q9**	30	1.00	Approved	30	1.00	Approved
**S-FVI/Ave**	0.95			0.93		

Ne ¹: Number of experts evaluated the items, Item-level face validity index (I-FVI): = (agreed item)/(number of raters), ≥0.80 approved; S-FVI/Ave = (sum of I-FVIscores)/(number of items).

## Data Availability

The data are not publicly available due to privacy and confidentiality. However, restrictions apply to the availability of hospital data and are available from the authors with the permission of the organization.
